# VasoTracker, a Low-Cost and Open Source Pressure Myograph System for Vascular Physiology

**DOI:** 10.3389/fphys.2019.00099

**Published:** 2019-02-21

**Authors:** Penelope F. Lawton, Matthew D. Lee, Christopher D. Saunter, John M. Girkin, John G. McCarron, Calum Wilson

**Affiliations:** ^1^Centre for Advanced Instrumentation, Biophysical Sciences Institute, Department of Physics, Durham University, Durham, United Kingdom; ^2^Strathclyde Institute of Pharmacy and Biomedical Sciences, University of Strathclyde, Glasgow, United Kingdom

**Keywords:** pressure myography, blood vessel function, smooth muscle, endothelium, vasodilation, contraction

## Abstract

Pressure myography, one of the most commonly used techniques in vascular research, measures the diameter of isolated, pressurized arteries to assess the functional activity of smooth muscle and endothelial cells. Despite the widespread adoption of this technique for assessing vascular function, there are only a small number of commercial systems and these are expensive. Here, we introduce a complete, open source pressure myograph system and analysis software, VasoTracker, that can be set-up for approximately 10% of the cost of commercial alternatives. We report on the development of VasoTracker and demonstrate its ability to assess various components of vascular reactivity. A unique feature of the VasoTracker platform is the publicly accessible website (http://www.vasotracker.com/) that documents how to assemble and use this affordable, adaptable, and expandable pressure myograph.

## Introduction

Alterations in the regulation of blood vessel diameter is either a primary determinant or a consequence of vascular diseases such as hypertension and diabetes (Mulvany et al., 1978; Durante et al., 1988; Kappagoda et al., 1989; White and Carrier, 1990; Li and Bukoski, 1993; Touyz et al., 1995). As such, the measurement of vessel caliber is often used to assess vascular function (Angelsen and Brubakk, 1976; Qamar et al., 1986; Lee et al., 2009). Conceptually, the relationship between blood flow and vessel diameter is simple. As blood vessel diameter increases, there is a decrease in the forces (e.g., friction) that resist the flow of blood and a net increase in blood flow. Conversely, a reduction in vessel diameter results in decreased blood flow. Though the theoretical relationship between vessel diameter and blood flow is straightforward, the behavior of the vasculature *in vivo* is not. *In vivo*, increases (vasodilation) and decreases (vasoconstriction) in blood vessel diameter result from a complex interplay of mechanical forces and vasoactive molecules originating in many different cell types. This innate complexity has led researchers to develop methods to study blood vessels in isolated, *ex vivo* preparations where many confounding factors can be controlled (Mulvany and Halpern, 1976; Duling et al., 1981; Halpern et al., 1984; Günther et al., 2010).

One methodology to measure artery diameter in isolated, *ex vivo*, artery preparations is pressure myography (Halpern et al., 1984). In the pressure myograph, isolated blood vessels are cannulated, connected to a pressure-perfusion system that controls intraluminal pressure, and the diameter of the artery is measured using microscopic techniques. A major advantage of using pressure myography to study blood vessel function is that the physiological, cylindrical configuration of the vessel wall is conserved, whilst confounding factors (e.g., neural and circulating influences) are eliminated. Moreover, minimal manipulation of the vessels is required (other than that necessary to excise the tissue) and many physiological responses that occur *in vivo* are retained. For example, isolated arteries develop spontaneous basal tone and display myogenic activity (Buus et al., 1994), and exhibit flow-mediated dilation (Kuo et al., 1990; Wilson et al., 2016a).

The simplicity, practicality, and fidelity of the pressure myograph for assessing artery function *ex vivo* is evidenced by its widespread adoption in vascular research labs. However, the majority of pressure myographs used in vascular research are commercial systems that are available from two principal suppliers (Danish Myo Technology, Denmark, and Living Systems Instrumentation, United States). The systems supplied by these companies are robust, well-documented devices but are rather expensive (Jadeja et al., 2015). At the time of writing, a complete commercial system can cost more than £40,000. This expense presumably reflects the low-volume production of scientific equipment, as pressure myograph systems consist of little more than a camera-attached microscope, pressure transducers, analysis software, and optionally, fluid pumps. Here, we describe the construction and use of a complete pressure myograph system (with heated myograph chamber, temperature controller, pressure head and pressure monitor, microscope, computer, and diameter analysis software) that can be set-up for as little as £3,500. Half of this cost arises from the purchase of a basic microscope and a computer. The design of the system follows open source principles and, as such, we make available a complete component list, design files, software, and instructions for building and operating the system/software. This follows the route taken by the OpenSPIM project (Pitrone et al., 2013), where the release of microscope blueprints has spurred on an entire community of researchers to build their own instruments (Girkin and Carvalho, 2018). In releasing VasoTracker as part of the open source movement, we hope to improve the accessibility of the pressure myograph and provide a platform (hardware and software) that will support the technical development of the technique for tailored experimental protocols.

## The Vasotracker Pressure Myography System

**FIGURE 1:**
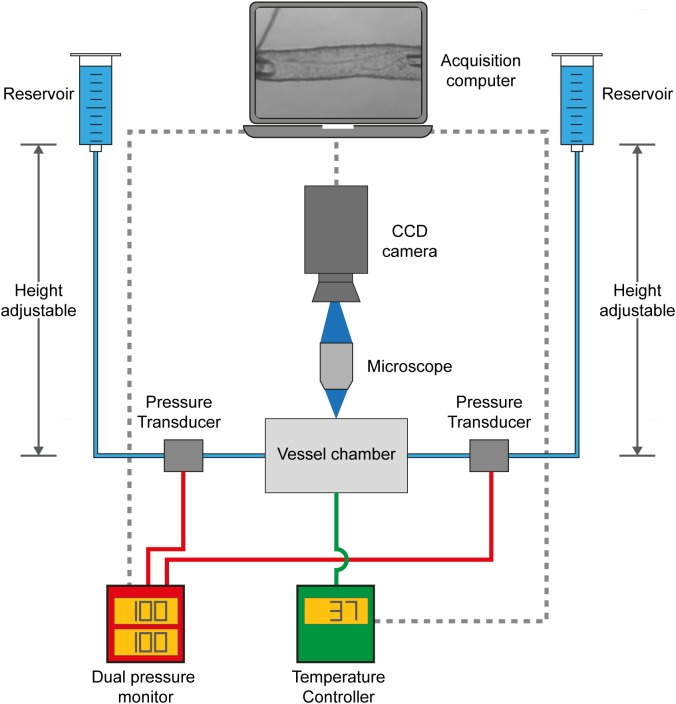
VasoTracker, an open source pressure myograph system. Schematic diagram showing the core components of VasoTracker. With the VasoTracker system, arteries are mounted in a custom vessel imaging chamber and imaged by a large-format CCD camera mounted on a microscope. Intraluminal pressure and flow are controlled via two height-adjustable reservoirs. Pressure is monitored by an Arduino pressure monitor. The imaging chamber temperature is controlled by another Arduino. Image, pressure, and temperature data is acquired, stored and displayed by bespoke acquisition software that automatically determines outer and inner blood vessel diameter.

VasoTracker (Figure 1) is a pressure myography system that provides measurement of outer diameter, lumen diameter, wall thickness, control and measurement of intraluminal pressure and temperature in a range of blood vessel sizes. In designing VasoTracker, we wanted to produce a system that might lower the cost of pressure myography and help expand the use of the technique in both research and teaching laboratories, whilst also increasing the flexibility of the method and enabling easier integration with other experimental approaches (e.g., other imaging techniques). To achieve this, we have, as much as possible, built VasoTracker using existing open source hardware and software solutions. Control electronics are based on open source Arduino microcontrollers and associated open source expansion boards (called “shields”) that extend the Arduino’s capabilities. The VasoTracker software is written in the open source programming language, Python (Python Software Foundation^1^), using libraries from the open source software for microscope imaging, μManager (Stuurman et al., 2010) The complete system includes a myograph bath chamber, temperature controller, pressure monitor, CCD camera, microscope, computer, and acquisition/analysis software. The complete component list, design files, software, and instructions for building and operating the system are available from the VasoTracker website and repository^2^^,^^3^. Data supporting the findings of this study are available for download with the VasoTracker software, or from the corresponding authors on request.

### VasoTracker Hardware Overview

The central hardware component of the VasoTracker system (Figure 1) is the VasoTracker vessel chamber, a custom imaging chamber in which a blood vessel may be mounted between two cannulae. Each cannula is connected to one of two height-adjustable reservoirs which permit easy adjustment of the intraluminal pressure. Experiments may be conducted with, or without, flow through the lumen of the vessel. Pressure is monitored by inline pressure transducers and an Arduino-based data acquisition system. The vessel chamber itself contains heating elements that enable experiments to be performed at physiological temperatures, avoiding the need for superfusion. Temperature is measured and controlled by an additional Arduino-based temperature controller. The VasoTracker bath sits on a microscope and blood vessels are imaged using a CCD camera attached to the microscope camera port. The VasoTracker software, installed on a basic laboratory computer, is used to display images of the mounted blood vessel and real time traces of vessel diameter (inner and outer), temperature and transmural pressure.

#### Vessel Chamber

**FIGURE 2:**
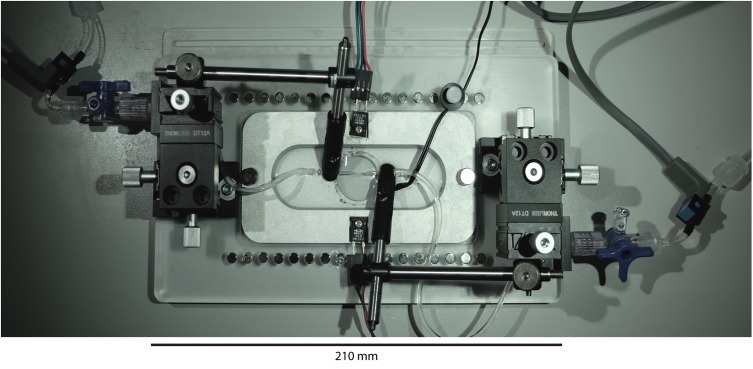
VasoTracker vessel chamber. Photograph of the vessel chamber containing an artery mounted on two glass cannula. The x-y-z linear position translators at either end of the bath (black boxes) provide very smooth and accurate cannula movement and alignment. The translators permit easy cannula movement even during an experiment.

The VasoTracker vessel chamber (Figure 2) is constructed from two components; a metal (aluminum) insert and an acrylic base (holder), which are held together by two thumb screws. The materials were chosen to provide efficient heat transfer to the bath solution via two resistive heating elements. Design files for the vessel chamber are provided on the VasoTracker website (and links therein), allowing full customization and free choice of manufacturing technique. We used a company specializing in CNC routing (Proto Labs Ltd., Shropshire, United Kingdom) to manufacture these parts. However, users are free to use to use local manufacturing options.

Mounted on each end of the chamber base are cannula holders (MSC-1 M, Siskiyou, OR, United States). The cannulae holders are held in place by 3-axis translation stages (DT12XYZ/M, Thorlabs, Newton, NJ, United States) that enable simple and precise axial alignment of the cannula and positioning of the blood vessel. A window, centered in the bottom of the chamber and sealed with a circular coverslip, permits light transmission and allows the chamber to be mounted on either upright or inverted microscopes. During the course of an experiment, drugs may be added to the chamber using pipettes. However, the base of the chamber is studded with neodymium magnets that allow perfusion (or oxygenation) plumbing to be held in place with magnetic holders (e.g., MAG-2 magnetic clamp, Warner Instruments, Hamden, CT, United States). Use of perfusion plumbing (such as bent 16G needles; NB16G1.5B90, NeedlEZ, Hoyland, United Kingdom) enables convenient replacement of the bath solution over the course of an experiment. The magnets are also used to hold the temperature sensor (epoxy-coated NTC thermistor) in place (see Figure 2).

#### Imaging System

VasoTracker uses a microscope (T610D, AmScope, Irvine, CA, United States) equipped with a large-format CMOS camera (DCC1545M, 1280 × 1024 pixels, 5.2 μm pixel size, Thorlabs, NJ, United States) to acquire images of pressurized blood vessels. The AmScope T610D is an economical, upright microscope model. Should users wish to use VasoTracker with an inverted microscope, relatively inexpensive models are available from a range of suppliers (e.g., Eclipse Ts2, ∼£3100; Nikon, Tokyo, Japan).

#### Temperature Controller

**FIGURE 3:**
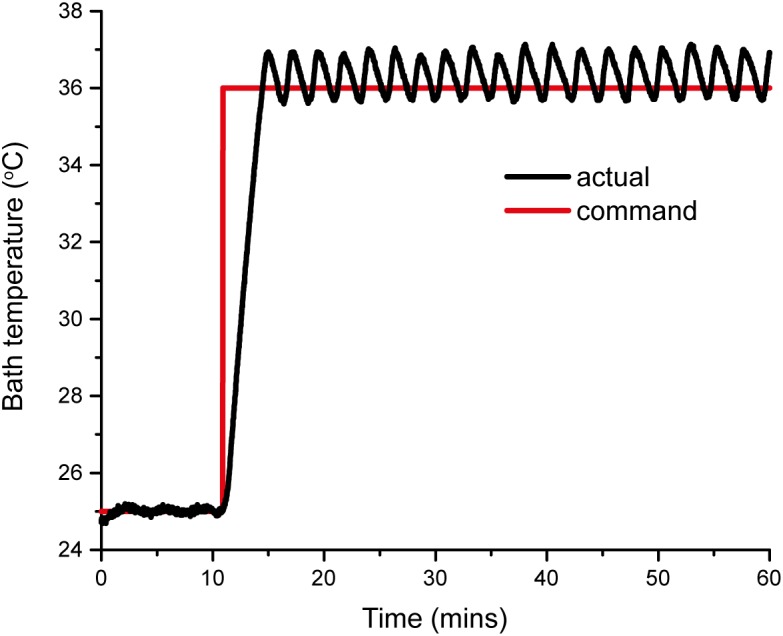
VasoTracker temperature controller response curve. The curves show the actual response (black line) to a step command from 25 to 37°C. The bath temperature reaches the set point in approximately 5 min and maintains this temperature with a stability better than ±1°C.

Pressure myography experiments are usually performed at physiological temperature (37°C) using either of two heating systems: (1) a superfusion system, in which the bath solution is heated externally (for example, by a water bath) and continuously (re)circulated through the bath chamber; or (2) using a built-in heating system that maintains the chamber temperature. To avoid the need for costly circulation pumps and an external heater, the VasoTracker system utilizes a built-in heating system. Two resistive heating elements (Kool-Pak 0.2Ω, Caddock, Riverside, CA, United States) are mounted on the imaging chamber base and the chamber temperature is monitored by a temperature sensor (10k NTC Thermistor, Adafruit, New York City, NY, United States) held in the chamber. The desired temperature is set by the user on the temperature controller. The controller uses a simple “bang-bang” control method that switches the heaters on/off when the bath temperature is below/above this desired temperature, as appropriate. This method of heating control achieves stability of better than ±1°C (Figure 3). The desired and measured temperature are displayed in real time on a local LCD display, and both are reported back to the VasoTracker analysis software via the Arduino serial port. The small temperature variations seen were not noticed to cause any changes in vessel diameter.

#### Pressure Head and Pressure Controller

To enable pressure to be applied to cannulated blood vessels, each cannula is connected to an independent reservoir (Kuo et al., 1990). For convenience, the VasoTracker system employs 20 ml syringes as the reservoirs, as each of these may be mounted to a steel rail via a magnetic coupling built from low-cost cheap optical components supplied by Thorlabs. The magnetic coupling enables the height of each of the reservoirs to be adjusted (independent of one another) by changing the position on a magnetic rail (steel bar), thus setting the hydrostatic pressure at the level of the artery in the chamber. Flow through the lumen can be achieved by offsetting the height of the two reservoirs, such that a pressure gradient is established. The pressure set up by each of the columns is monitored by flow through pressure transducers (26PCDFG5G, Honeywell, Morris Plains, NJ, United States) and a low-cost Arduino data acquisition system consisting of an Arduino Uno, a Wheatstone bridge shield (RB-Onl-38, RobotShop, Mirabel, QC, Canada) and an LCD display shield (LCD 1602, iTead Studio, Shenzen, China). The VasoTracker pressure monitor displays the pressure measured by each transducer in real time on the LCD display and reports back to the VasoTracker analysis software via the Arduino serial port.

### The VasoTracker Software

The VasoTracker software was created, first and foremost, as an interface for monitoring pressure myography experiments in real-time. As such, the VasoTracker software performs four main functions:

(1) Acquisition, display, and recording of images of pressurized blood vessels from the microscope-attached digital camera.
(2) Acquisition and recording from the Arduino temperature and pressure control systems.
(3) Real time calculation, graphing, and recording of blood vessel diameter.
(4) Monitoring of user interventions (e.g., the addition of biological compounds to the vessel chamber).

The software was written entirely in the Python (2.7) programming language, with camera control libraries provided by μManager (Stuurman et al., 2010).

#### The VasoTracker GUI

**FIGURE 4:**
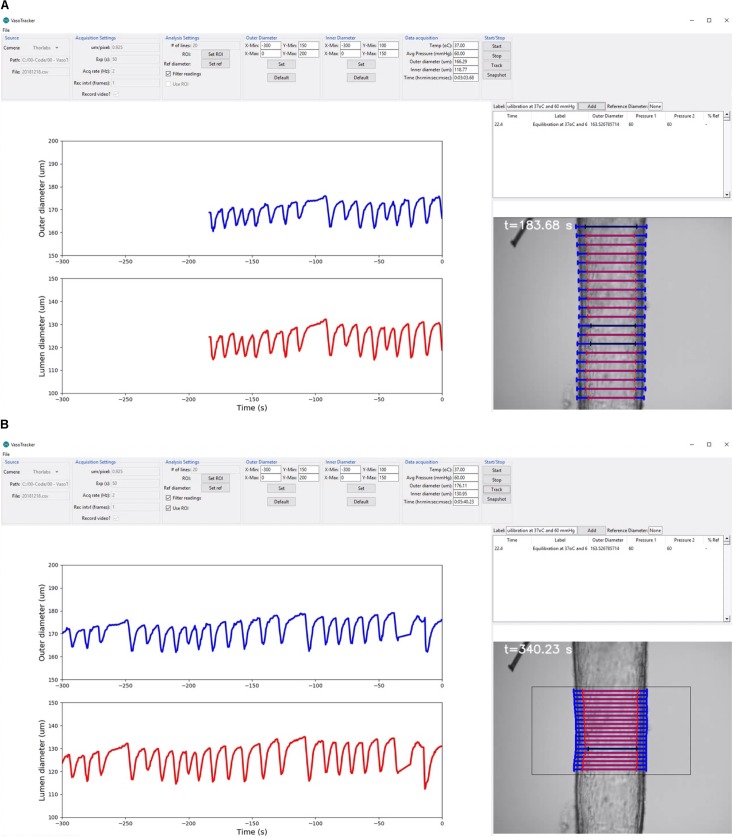
VasoTracker software. **(A,B)** Views of the VasoTracker graphical user interface during an experiment in which a posterior cerebral artery (from a male rat) developed spontaneous vasomotion at 60 mmHg. **(A)** Shows outer diameter (blue lines) and inner diameter (red lines) measurements determined for 20 scan lines spaced vertically along the longitudinal axis of the artery. **(B)** Shows the same artery at a later time point after the diameter measurement has been restricted to a user-defined region of interest. In these examples, the graphs show average diameter measurements for the most recently acquired 300-s period, with the most recently acquired data point shown at *t* = 0 s (i.e., the data moves to the left as new data is acquired). Outlier diameter measurements are detected using the modified z-score, are excluded from the average diameter calculation, and are overlaid in black on the image display. The full dataset from which these screenshots were taken is shown in Supplementary Video S1.

The main purpose of the graphical user interface (GUI) is to display, in real-time, the diameter of pressurized blood vessels mounted in the myograph vessel chamber. The user interface consists of four main elements (Figure 4):

(1) A control panel, positioned at the top of the main application window, containing widgets that enable users to view/change the VasoTracker settings. This control panel is composed of seven groups: (1) the source group, where the camera is specified and which displays the current file directory; (2) the acquisition settings group, where camera settings (pixel scale factor, exposure) and image export (.tiff format) settings are configured; (3) the analysis settings group, where the number of scan lines used to determine vessel diameter is set, and which contains buttons to enable a region-of-interest (ROI, to limit the field-of-view in which vessel diameter is determined) and to set a reference diameter, and choose whether or not to filter measurements using the ROI or algorithmically; (4 and 5) the outer diameter and an inner diameter groups, where the graph display settings can be modified; (6) the data acquisition group, where the current temperature, pressure, outer diameter, inner diameter, and time are displayed; and (7) the start/stop group, where image acquisition and diameter analysis can be enabled/disabled, and which contains a button to take a snapshot image of the current field-of-view.
(2) A live updating graph panel, which displays both inner and outer diameter measurements, each on one of two auto-scrolling graphs. The scrolling graphs show the most recently acquired data, with the newest plotted on the right (at *t* = 0 s). As each new data point is acquired, the previously plotted data is shifted to the left. When the lower axis limit is reached (defaults of *t* = -600 s), the graph continues to display only the most recently acquired data. However, all data is stored in memory and the axis limits are adjustable (set in the outer/inner diameter control panels), allowing the full experimental time-course, or a specific section of the time-course, to be visualized at any time.
(3) A real-time image-feed, displaying the microscope field-of-view on screen with diameter indicators overlaid.
(4) A data entry table, where experimental treatments (e.g., drug additions) can be logged. When notes, which may be typed into a text entry box, are added, the table is automatically populated with the current time, diameter and pressure measurements. Additionally, if a reference diameter has been set (by pressing the “Set Ref” button in the analysis settings panel), the current diameter is converted to a % (of the reference diameter) to provide the user with a convenient measure of arterial tone.

#### Blood Vessel Diameter Analysis Algorithm

**FIGURE 5:**
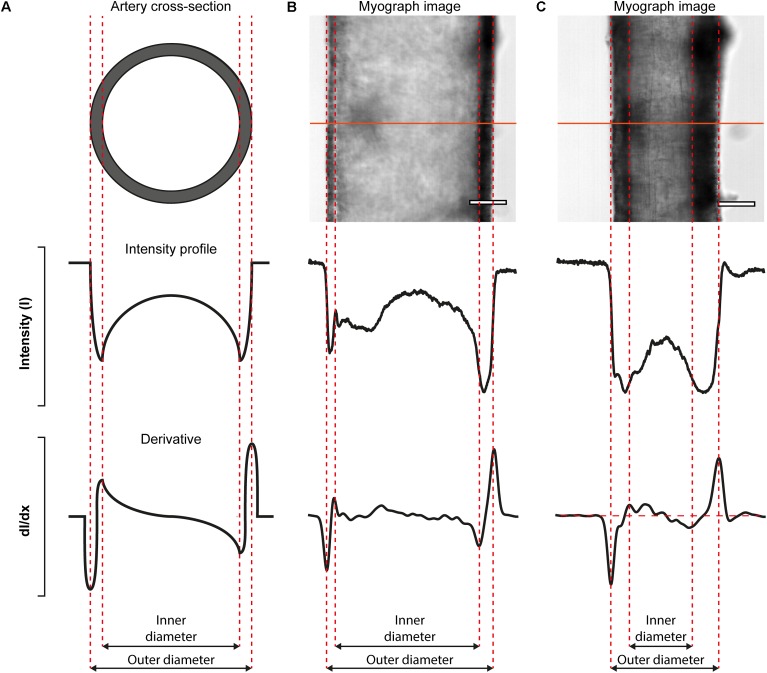
Algorithmic determination of inner and out diameter of pressurized blood vessels. **(A)** Schematic diagram (cross-sectional view, top) of an artery, idealized intensity profile (middle), and the first derivative of the idealized intensity profile (bottom) illustrating how peaks in the derivative profile may be used to identify the edges of an artery. **(B,C)** Real-data examples illustrating the algorithm. The top panels show a first-order mesenteric artery (male rat), pressurized to 80 mmHg, in the absence **(B)** and presence **(C)** of the α-adrenergic agonist, phenylephrine (1 μM). Scale bars = 100 μm.

The VasoTracker system takes advantage of variations in intensity that are present in images of pressurized blood vessels (Figure 5). These variations arise because the artery is held orthogonal to the *z*-axis of the microscope system such that only the mid-plane of the artery is in focus. When imaged in this way, alterations in the optical density of the vessel, which manifest as changes in the intensity profile, permit the wall of the artery to be easily identified. Figure 5 illustrates the concept for calculating vessel diameter employed by the VasoTracker software. Edges of the blood vessel correspond to rapid changes in light intensity profiles that are measured perpendicular to the long axis of the blood vessel (scan lines). VasoTracker identifies these rapid changes by detecting peaks in the derivative of the intensity profile (integrated across 25 pixels). Vessel diameter is calculated for a default number, 20 (which can be changed), of equally spaced scan lines along the length of the artery, and averaged to give a reliable measure of outer and inner diameter. For each of the scan lines, outer and inner diameter are indicated on the real-time image feed indicated by a blue or red line, respectively.

In cases where the presence of side-branches, adherent fat/connective tissue, or debris cannot be avoided, the tracking algorithm may fail to accurately track the vessel wall. VasoTracker has two useful features that may help minimize errors in these circumstances. First, the modified z-score test (Iglewicz and Hoaglin, 1993) can be used to identify and exclude outliers from the average diameter calculation. If this option is enabled (“Filter data” check box in Analysis Settings), outliers are indicated on the live image feed by black coloring. Second, users may specify a region of interest that avoids side-branches or adherent fat/connective tissue which VasoTracker will then use to track diameter.

#### Data Acquisition Specifications

##### Image size

**FIGURE 6:**
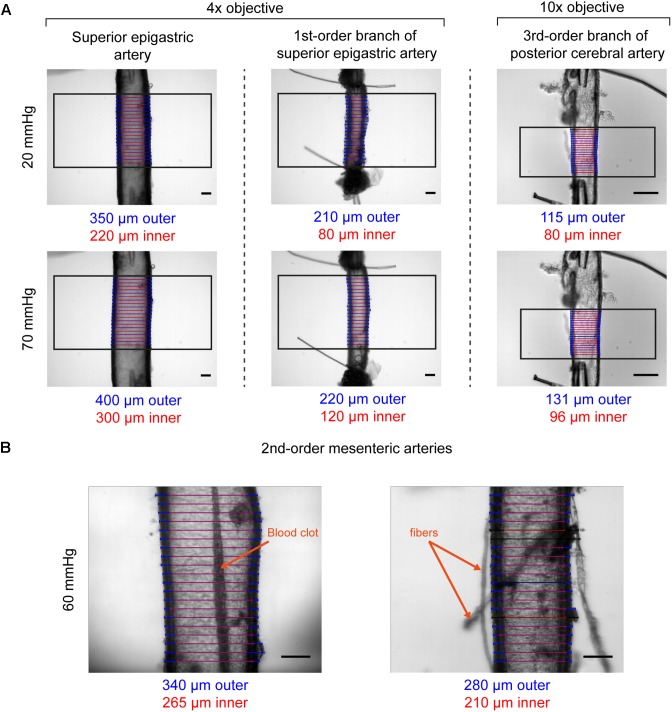
Examples of VasoTracker diameter detection. **(A)** Example images showing outer and inner diameter tracking in the superior epigastric artery, a first-order branch of the superior epigastric artery, and a third-order branch of the posterior cerebral artery. Each artery (obtained from male rats) was exposed to 20 mmHg and 70 mmHg intraluminal pressure, and the diameter tracking algorithm was confined to a region of interest (black box). Blue/red scan lines indicate outer and inner diameter measurements, respectively. Outliers are colored black. **(B)** Example images showing diameter tracking in second-order mesenteric arteries (from male rats) pressurized to 60 mmHg imaged under sub-optimal conditions. The artery shown on the left has a blood clot in the middle of the lumen which could not be flushed out. The artery on the right has numerous fibers and other debris attached to the adventitia. Despite the condition of these arteries, in both examples VasoTracker is quite capable of accurately tracking the vessel wall. All image scale bars = 100 μm.

The T610D microscope comes equipped with two objectives (4× and 10× magnification) that can be used to visualize arteries mounted in the vessel chamber. Coupled with the recommended camera, the objectives provide fields of view of 1.65 mm by 1.33 mm (4× magnification, 1.3 μm projected pixel size) or 0.66 mm by 0.53 mm (10× magnification, 0.52 μm projected pixel size). The objectives permit vessels with diameters ranging from <100 μm to ∼1.55 mm (see Figure 6A and figures thereafter). Depending on the image contrast, diameter can be successfully tracked even if vessels contain blood clots, or if debris attached to the artery wall (Figure 6B). If required, the microscope magnification (and hence the field of view and resolution) can be altered further using alternative objectives and/or coupling lenses between the camera and the microscope.

##### Acquisition rate

Two factors affect the achievable sampling rate: image quality, and processing speed. To achieve optimal diameter tracking, images should utilize the full dynamic range of the camera (e.g., images should be grayscale, and not either very dark or very bright). The present release of the VasoTracker GUI (version 1.0.1) enables the camera exposure to be adjusted (between 10 ms and 500 ms), thus enabling the optimization of images. We find that, with an exposure of 10 ms, the recommended camera (Thorlabs DCC1545M) can obtain excellent images even under low illumination intensities, thus potentially permitting acquisition speeds up to 100 Hz. However, as of VasoTracker 1.0.1 (the version released at the time of publication), the sampling rate is set to 2 Hz (irrespective of camera exposure). This limit has been imposed to provide an adequate buffer for processing and graphing the resulting data.

#### Data Export

The VasoTracker software exports data in .csv format, which can be easily imported into all common data analysis packages. Each experimental run results in three .csv files: the first .csv file contains a record of time, temperature, pressure measurements from each of the two pressure transducers, the mean pressure experienced by the blood vessel, and the mean outer/inner diameter measurements; the second .csv file second contains the diameter measurements for each of the scan lines; and the third .csv file contains the experimental data input into the data entry table (e.g., notes made by the investigator, drug additions, etc.). Additionally, the user may choose to record the blood vessel images. If the “Record Video” checkbox (located in the Acquisition Settings control panel) is ticked, images (with and without diameter overlays) will be saved at a user-defined interval (by default every frame is exported) in .tiff format.

## Pressure Myograph Experiment Verification

The pressure myograph is a flexible system that can be used to assess various properties of blood vessels. To demonstrate the effectiveness and versatility of VasoTracker, the records of several experiments that we have undertaken since establishing the system are described below.

### Agonist-Evoked Contraction and Dilation

**FIGURE 7:**
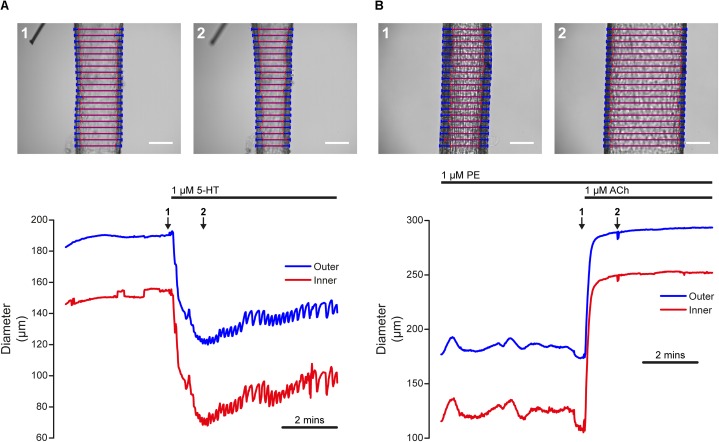
Dynamic tracking of artery diameter during agonist-evoked contraction and relaxation. **(A)** Time-course of contraction of a posterior cerebral artery (60 mmHg) in response to 5-HT (1 μM). The top panels show the artery before (1) and after (2) the application of 5-HT. The lower panel shows the diameter measured by VasoTracker (average of 20 line-scans). Outer (blue) and inner (red) diameter are shown. The time points corresponding to the images shown in the upper panel are indicated by the numbered arrows. **(B)** Time-course of dilation of a pre-contracted (PE, 1 μM) second-order mesenteric artery (60 mmHg) to ACh (1 μM). Images show the artery before (top, 1) and after (top, 2) the application of ACh (1 μM). Again, the bottom panel shows average artery diameters (outer and inner, 20 line-scans) measured by VasoTracker for the full experiment. Both arteries obtained from male rats. Scale bars = 100 μm.

Artery smooth muscle cell function is frequently assayed by measuring blood vessel contraction to Ca^2+^-mobilizing agonists that act on receptors. Such an experiment, performed using VasoTracker, is illustrated in Figure 7A. The upper panel of Figure 7A show images of a rat posterior cerebral artery (McCarron and Halpern, 1990) pressurized to 60 mmHg (initial outer diameter = 185 μm, initial inner diameter = 147 μm), before (left) and after (right) the application of 5-HT (1 μM) to the chamber. The lower panel of Figure 7A shows the time course of the initial 5-HT-induced contraction and the subsequent development of vasomotion over a period of 6 min. The time points labeled 1 and 2 in the lower panel correspond to the images 1 and 2 in the upper panel. During this recording, average inner and outer diameter were calculated for the default number of line-scans (20). The results of the tracking algorithm are shown overlaid on the original images and it is evident from these that VasoTracker is able to accurately track both the inner and outer artery wall.

The functionally opposite response to contraction is dilation (an increase in blood vessel diameter). Many stimuli initiate dilation by acting on endothelial cells to stimulate the release of nitric oxide or the production of endothelial-derived hyperpolarization (Palmer et al., 1987; Taylor and Weston, 1988). Damage to the endothelium may impair or even abolish these responses (Furchgott and Zawadzki, 1980). Thus, endothelial function is often assayed by measuring the relaxation effects of chemical or mechanical mediators acting on the endothelium of pre-contracted blood vessels. An example of endothelium-dependent dilation, performed in the pressure myograph, is shown in Figure 6B. The upper panels of Figure 7B show images of a pressurized (60 mmHg), pre-contracted (phenylephrine, 1 μM) second-order rat mesenteric artery (McCarron et al., 1991) before (left) and after (right) the application of a supra-maximal concentration of the endothelial-dependent vasodilator, acetylcholine (ACh, 1 μM).

### Myogenic Reactivity

**FIGURE 8:**
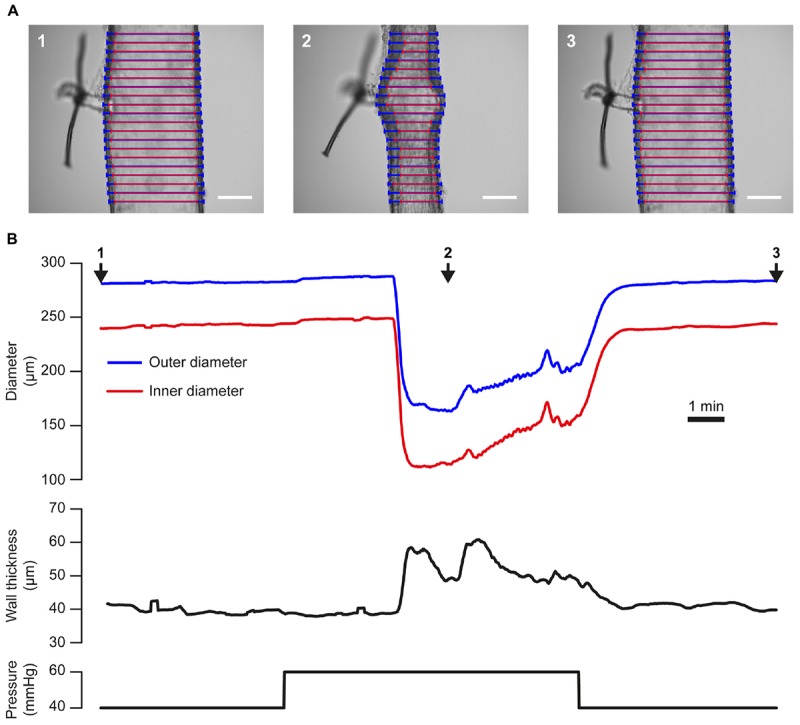
Tracking artery dimensions in blood vessels that exhibit myogenic tone. **(A)** Images of a single posterior cerebral artery from a male rat taken during an experiment in which the vessel was subject to a step-wise increase [from 40 mmHg (1), to 60 mmHg, (2)] then a decrease [to 40 mmHg, (3)] in pressure. A ∼20 μm diameter side branch, which was tied off with a single strand of thread, can be seen protruding from the vessel. Blue and red lines indicate VasoTracker measurements of outer and inner vessel diameter, respectively. Scale bars = 100 μm. **(B)** Traces showing the full time-course of outer and inner diameter (top), wall thickness (middle), and pressure (bottom) for the experiment shown in **(A)**. Numbered arrows indicate the time corresponding to the images in **(A)**. Upon increasing transmural pressure from 40 to 60 mmHg, the artery immediately dilated and subsequently developed myogenic tone and contracted to ∼50% of its diameter at 40 mmHg. Decreasing transmural pressure back to 40 mmHg caused a dilation of the artery back to the initial diameter.

Changes in blood vessel diameter may also be induced by mechanical forces acting on the vessel wall. The use of the VasoTracker system to study one such response (myogenic reactivity) is illustrated in Figure 8. In this example, a rat posterior cerebral artery with a small side-branch (∼20 μm diameter) is shown (Figure 8A). The side branch was tied off using a single strand of thread to prevent intraluminal flow. A stepwise increase in intraluminal pressure from 40 to 60 mmHg (Figure 8B, lower panel), evoked a small passive dilation (from 283 μm to 288 μm, outer diameter, Figure 8B, upper panel), followed by a contraction to a level significantly below baseline (164 μm, outer diameter). Conversely, when pressure was dropped back to 40 mmHg, the vessel relaxed back to its initial diameter. The ability of many small arteries to respond to a pressure increase by contracting (and to a pressure decrease by dilating) is independent of any neural or humoral influences, is termed the myogenic response (Meininger and Davis, 1992), and may be critical to the establishment of basal vascular resistance and the regulation of blood flow.

### Propagated Vasodilation

**FIGURE 9:**
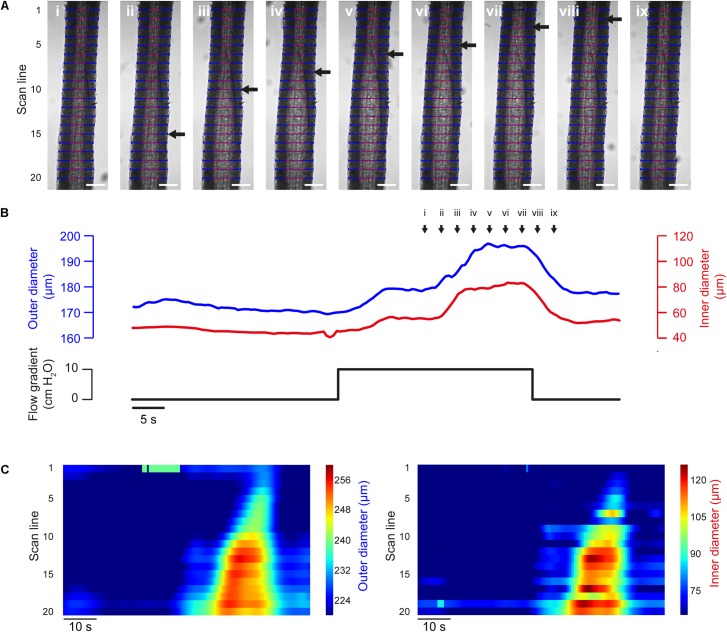
Flow-induced propagated vasodilation **(A)** Images of a pre-contracted (PE, 500 nM) third-order rat mesenteric artery (male rat) at nine time points during an experiment in which dilation was initiated by intraluminal flow (∼100 μl/min, established by a 10 cm H_2_O pressure gradient across the vessel). After an initial partial dilation, another dilation occurred. The second dilation propagated along the length of the artery (wave front indicated by black arrow). Blue and red lines indicate VasoTracker measurements of outer and inner vessel diameter, respectively. Scale bars = 100 μm. **(B)** Traces showing average outer and inner diameter measurements (top) and the pressure gradient (bottom) for the experiment shown in **(A)**. **(C)** Heat plots showing diameter (color) plotted against time (*x*-axis) for each of the scan lines (*y*-axis) shown in **(A)**. The propagated nature of the vasodilation is evident in the contours of the heat plot.

The propagation of vasodilation is critical to the coordination of arterial tone (Duling and Berne, 1970; Segal and Duling, 1986; Segal, 2015) and can be captured by VasoTracker (Figure 9). Subsequent analysis using the diameters determined at each individual scan line and output by VasoTracker can be used to demonstrate the progression of the dilation.

### Passive Properties of Blood Vessels

**FIGURE 10:**
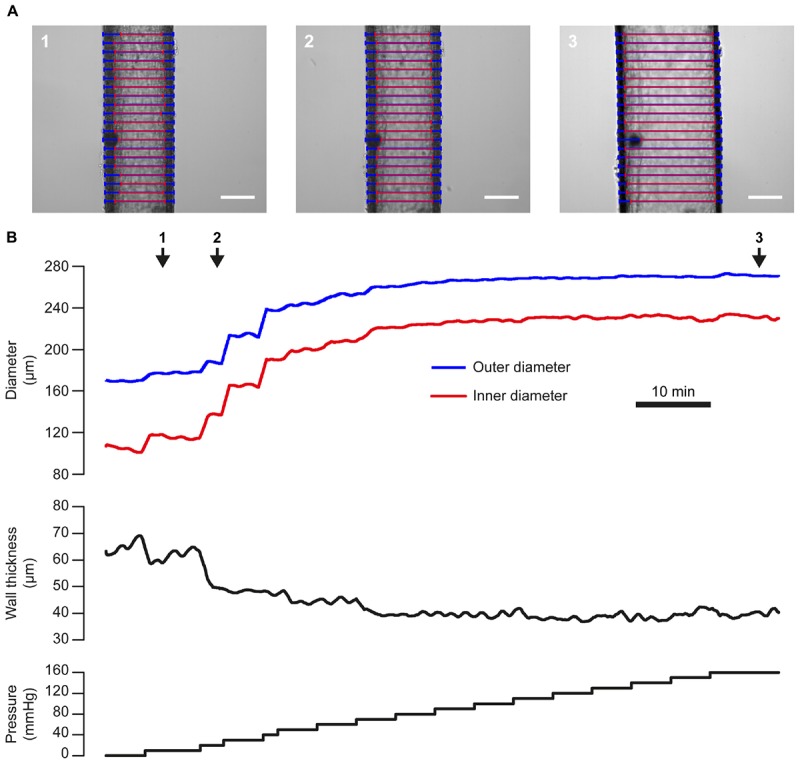
Pressure-diameter relationship. **(A)** Images of a single third-order mesenteric artery (male rat) at three time points during an experiment in which the vessel was subject to a step-wise increases in pressure from 0 to 160 mmHg (in a Ca^2+^-free bath solution). Blue and red lines indicate VasoTracker measurements of outer and inner vessel diameter, respectively. Scale bars = 100 μm. **(B)** Traces showing the full time-course of outer and inner diameter (top), wall thickness (middle), and pressure (bottom) for the experiment shown in **(A)**. Numbered arrows indicate the time corresponding to the images in **(A)**.

The response to mechanical stimuli may depend on both passive and active properties of the vascular wall. The passive properties of blood vessels may be assessed by studying the relationship between pressure and diameter in a Ca^2+^-free bath solution, as shown in Figure 10. This pressure-diameter relationship largely results from the passive mechanical properties of the main constituents of the vascular wall (smooth muscle cells, elastin, collagen) and is often used to assess arterial remodeling (e.g., Bakker et al., 2006).

### Comparison With a Commercial Alternative

**FIGURE 11:**
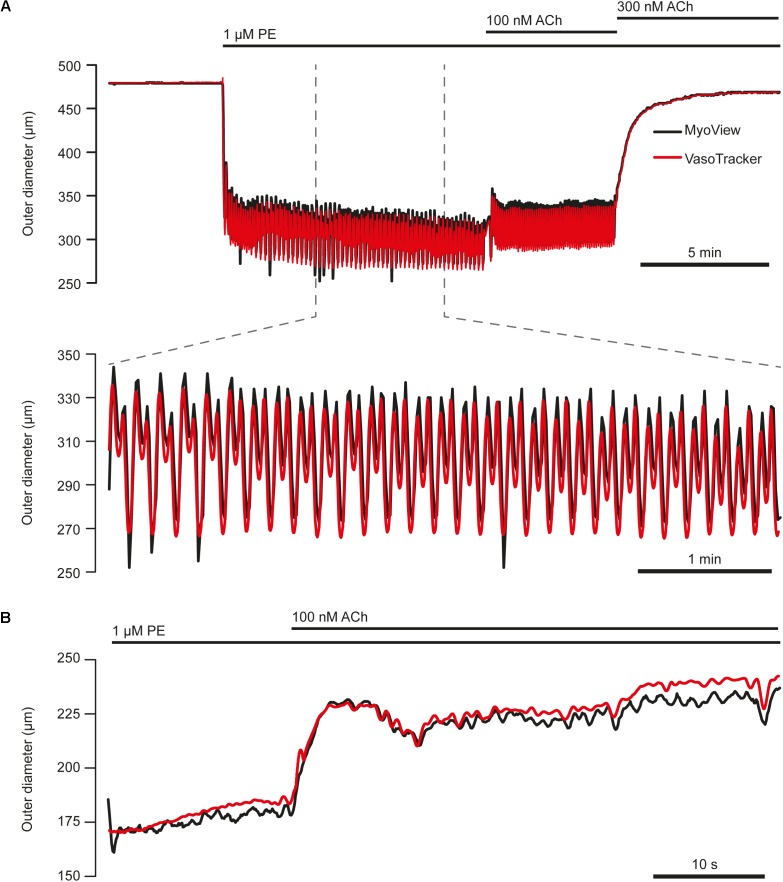
Comparison of VasoTracker diameter tracking performance with commercial software. **(A)** Top: full time course of an experiment in which the outer diameter of a first-order mesenteric artery (60 mmHg) from a male rat was tracked by both VasoTracker (red) and MyoView (black, Danish Myo Tech). Upon the addition of phenylephrine (1 μM) to the bath, the artery contracts and develops vasomotion (shown on expanded time course in bottom panel of **(A)**. ACh (100 nM) resulted in a slight dilation, whilst a slightly higher concentration of ACh (300 nM) abolished vasomotion and fully reversed the PE-induced contraction. **(B)** Example trace (VasoTracker, red; MyoView, black) showing the relaxation of a pre contracted (PE, 300 nM) third-order mesenteric artery (male rat) in response to ACh (100 nM).

To verify the tracking capability of VasoTracker, we performed experiments using a commercial pressure myograph system (Model 110P; Danish Myo Technology) and compared the results with those of the VasoTracker algorithm. In these experiments, arteries were mounted in the Danish Myo Technology myograph chamber and visualized at 10× magnification on an inverted Nikon Diaphot microscope. Images of the arteries were obtained by a CCD video camera (Watec, WAT-902A) and relayed to a computer for online diameter measurement and graphing (performed by the vessel tracking software, MyoView). MyoView software does not record the image feed. Thus, to enable a comparison with VasoTracker to be made, the camera feed was split and fed to a USB frame grabber (Dazzle DVC 100, Pinnacle Systems, Mountain View, CA, United States) and recorded on a separate computer system by μManager software. Images were recorded for subsequent off-line analysis by the VasoTracker algorithm. We found that VasoTracker measurements matched those obtained by the MyoView algorithm (Figure 11).

## Discussion

In many areas of biological research, researchers are harnessing the open source methodology to replace closed source commercial devices and software with flexible alternatives. The open source principle has created a catalog of system and software designs (which can be readily modified) to assist researchers in their scientific endeavors. For example, researchers can now furnish their laboratory with open source syringe pumps (Wijnen et al., 2014), PCR machines^4^, pulse generators (Sanders and Kepecs, 2014), pressure ejection systems (Forman et al., 2017), and even microscopes (Holm et al., 2014; Sharkey et al., 2016; Chagas et al., 2017). Open source technologies offer the benefit of being easy to modify (thanks to transparent documentation) and as a result can often be adapted to meet particular specifications better than commercial alternatives. Additionally, open source software is available at no cost, and open source instrumentation may be obtained significantly cheaper (as designs are made freely available) than commercial alternatives. In the field of vascular physiology, there are numerous examples of open source software packages making an impact: Micro-Manager Open Source Microscopy Software (Edelstein et al., 2014), is used routinely by vascular physiologists for image acquisition (e.g., Devraj et al., 2016; Wilson et al., 2016b); as are ImageJ and FIJI (Schindelin et al., 2012; Rueden et al., 2017) for image processing applications (Francis et al., 2012; Wilson et al., 2016c; Sabbagh et al., 2018). However, there are limited open source hardware options available for the vascular physiologist to exploit.

Here, we add to the catalog of open source tools an inexpensive and flexible pressure myography system, VasoTracker, which permits the vascular activity of isolated, pressurized blood vessels to be monitored. VasoTracker can be used to investigate smooth muscle cell and endothelial cell function under a wide variety of experimental conditions. The system includes all components that would be expected from a commercial pressure myograph system, but at a fraction of the cost (∼10%): a vessel chamber for mounting arteries; pressure columns and a pressure monitor for establishing and monitoring intraluminal pressure; heaters and a temperature controller for controlling the chamber temperature; a microscope and CCD camera for imaging; and software for acquiring images and calculating blood vessel diameter. VasoTracker has been assembled with the open source paradigm (Pearce, 2012) in mind and, as much as possible, makes use of existing hardware and software open source solutions. For example, both the pressure monitor and the temperature controller are built using Arduino electronics boards and the software was written entirely in Python.

By releasing VasoTracker under an open source license, we make the system and associated software available to any researcher. Complete instructions for building and operating the VasoTracker system and the VasoTracker acquisition software are available from the VasoTracker website (see footnote 2). The only costs associated with establishing the system is the time required to assemble the components and the cost of the components themselves. Assembling the system requires little technical expertise, and can be completed in less than 1 day by novice users. Installing the software is also straightforward, as it is managed by a single installation file. Those interesting in viewing or adapting the software to specific experimental questions may do so by downloading the source code. Much of the cost of the VasoTracker system arises from the purchase of a microscope and a dedicated computer, which may be readily available within the laboratory. We therefore recommend that, if possible, any disused microscopes are repurposed for use with the VasoTracker myograph components (the first iteration of the VasoTracker system was built on a disused 160 mm tube-length Nikon TMS-F microscope).

Pressure myographs were originally developed in research labs to answer particular questions about the regulation of blood vessel diameter (Halpern et al., 1984). As new questions arose, researchers devised modifications of the original design and a number of variations of the pressure myograph are now documented. In some myographs, one end of an artery is mounted on a cannula with the other end occluded (Halpern et al., 1984). In others, the artery is mounted on two cannula (Halpern and Osol, 1986). In another variation on the mounting technique, special double-barreled cannula are used to mount the arteries (Duling et al., 1981; VanBavel et al., 1990). Transmural pressure may be established by one of two techniques: either by a pressure head where reservoirs of physiological saline are set at a height to create a pressure column and so set the hydrostatic pressure, or by a servo-controlled pump system. Together, the many myograph variants permit the study of arteries across a range of sizes and under a range of conditions and, for example, permit the effects of flow at constant pressure be studied (Tesfamariam et al., 1985; Garcia-Roldan and Bevan, 1990; Kuo et al., 1990, 1991). The capabilities of the pressure myograph can be further extended by combining it with fluorescence techniques to assess arterial diameter (VanBavel et al., 1990; Falcone et al., 1993; Garland et al., 2017). The technique has even been adapted to enable visualization of leukocyte adhesion to the endothelium (Michell et al., 2011) and, more recently, to permit drugs to be applied to only half of the length of a single artery, allowing conducted responses to be assessed in the other half (Palao et al., 2018).

In addition to variation in the design of pressure myography systems, there is also substantial variation in the methods used to measure arterial diameter. The most basic form of diameter measurement is that performed manually by the experimentalist using video calipers (Devaney et al., 1976; Hogan et al., 1984; Pries and Gaehtgens, 1987; Goodman, 1988). However, because of the need for trained experimentalists to continuously monitor the equipment and make manual adjustments, automated methods have been devised (Wiederhielm, 1963; Intaglietta and Tompkins, 1972; Gotoh et al., 1982; Halpern et al., 1984; Neild, 1989). A number of these have been or are available commercially, but are video-based methods that utilize out-of-date equipment (i.e., analog cameras and AV equipment). As a result, more recent methods have utilized digital imaging and software based analysis methods (Fischer et al., 1996). Of these, MyoView (Liu et al., 2004; Rivera de los Arcos et al., 2004), the ImageJ plugin Vessel Diameter (Fischer et al., 2010), VesselTracker (Davis, 2005), MyoTracker (Fernández et al., 2014), and Mary (Edvinsson et al., 2007; Erdling et al., 2017) are routinely used by vascular physiologists.

The present release of VasoTracker is capable of tracking the diameter of pressurized arteries under a range of physiological conditions. However, the system could easily be adapted to improve its functionality and, ultimately, the ability of the system to help understand blood vessel physiology. For example, the temperature control system could be upgraded to implement PID temperature control. Closed-loop pressure/flow control could be achieved with two peristaltic pumps (one on the inflow and one on the outflow). Pressure control could be automated by implementing a closed-loop pressure-driven flow control scheme (Heo et al., 2016). VasoTracker could also easily be used with other existing, complementary research techniques. The availability of open source electrophysiology tools (Sanders and Kepecs, 2014; Sheinin et al., 2015) present an attractive option for adding electrical stimulation functionality to VasoTracker. The VasoTracker imaging chamber could even be redesigned so as to permit tissue culture experiments (Straub et al., 2012). In its present form, the system is compatible with any microscope. Thus, functionality could be increased by placing the VasoTracker chamber on the stage of one of many open source microscopes (e.g., Holm et al., 2014; Sharkey et al., 2016; Chagas et al., 2017). In this way, the system could easily be incorporated into existing fluorescence microscopes to permit the study of fluorescent dyes loaded into cells of the vessel wall (e.g., Ca^2+^ indicators, Lee et al., 2018, or mitochondrial probes, Durand et al., 2018; Wilson et al., 2018).

Though the diameter tracking performance of VasoTracker is comparable to a commercial alternative (Figure 11), the detection algorithm could be improved. For example, detection of the artery wall using the rate of change of image intensity (as in VasoTracker) sometimes fails to accurately track the inner vessel wall. Failure to accurately track the vessel wall may, among other reasons, occur because of irregular structures in specific regions of the vessel wall or if there is debris stuck to the artery. VasoTracker provides the ability to measure diameter at up to 50 separate scan lines, and uses a statistical filtering process to minimize the contribution of such inaccurate measurements to the overall (average) diameter measurement. In addition, an ROI may also be used to limit readings to specific areas of a blood vessel. Nevertheless, improved accuracy may be achieved by combining the edge detection algorithm with a lower limit on the thickness of the vessel wall (Fischer et al., 1996). The software may also benefit from the use of a model-based approach to vessel image analysis (Michoud et al., 1993; Smith et al., 1996; Crabtree and Smith, 1999).

## Conclusion

In conclusion, we have developed a flexible, open source pressure myograph system, VasoTracker, that can be setup for approximately 10% of the cost of commercial alternatives. It is capable of integration into microscopes already in use in many laboratories. The low entry barrier and the potential for future developments opens considerable potential for researchers to expand and enhance myography with tailored vascular reactivity experiments. We also hope that VasoTracker will find a home in the teaching laboratory in which pressure myography has traditionally been too expensive an option.

## Ethics Statement

All experiments described in this study used arteries obtained from male Sprague-Dawley rats killed by cervical dislocation. All animal studies were conducted with ethical approval from the University of Strathclyde Animal and Welfare Ethical Review Committee and in accordance with the United Kingdom Home Office regulations [Animals (Scientific Procedures) Act, 1986, United Kingdom].

## Author Contributions

CW, CS, JG, and JM developed the concept. CW, ML, PL, and CS wrote the software. CW and ML designed the experimental apparatus, performed the experiments, and analyzed the data. CW drafted the manuscript. CW, ML, CS, JG, and JM revised and edited the manuscript. All authors approved the final version of the manuscript.

## Conflict of Interest Statement

The authors declare that the research was conducted in the absence of any commercial or financial relationships that could be construed as a potential conflict of interest.

## Footnotes

1 https://www.python.org/

2 http://www.vasotracker.com/

3 https://github.com/VasoTracker/VasoTracker

4 https://openpcr.org/

## Supplementary Material

VIDEO S1 VasoTracker diameter tracking software. Screen recording of the VasoTracker software during an experiment in which a posterior cerebral artery (from a male rat) developed spontaneous vasomotion at 60 mmHg. Initially, diameter measurements are taken from the full length of the artery. Part of the way through the video, the measurements are restricted to a user-defined region-of-interest. Data shown in Figure 4.
